# Hyperforin Exhibits Antigenotoxic Activity on Human and Bacterial Cells

**DOI:** 10.3390/molecules22010167

**Published:** 2017-01-21

**Authors:** Petronela Imreova, Jana Feruszova, Stanislav Kyzek, Kristina Bodnarova, Martina Zduriencikova, Katarina Kozics, Pavel Mucaji, Eliska Galova, Andrea Sevcovicova, Eva Miadokova, Ivan Chalupa

**Affiliations:** 1 Department of Genetics, Faculty of Natural Sciences, Comenius University, Mlynská dolina B1, Ilkovičova 6, 842 15 Bratislava, Slovakia; petra.imreova@gmail.com (P.I.); jana.feruszova@gmail.com (J.F.); stanislavkyzek@gmail.com (S.K.); bodnarova.kristina@gmail.com (K.B.); sevcovicova@fns.uniba.sk (A.S.); miadokova@fns.uniba.sk (E.M.); 2 Cancer Research Institute, Biomedical Research Center, SAS, Dúbravská cesta 9, 845 05 Bratislava, Slovakia; martina.zduriencikova@savba.sk (M.Z.); katarina.kozics@savba.sk (K.K.); ivan.chalupa@savba.sk (I.C.); 3 Department of Pharmacognosy and Botany, Faculty of Pharmacy, Comenius University, Odbojárov 10, 832 32 Bratislava, Slovakia; mucaji@fpharm.uniba.sk

**Keywords:** hyperforin, antigenotoxicity, MTT assay, comet assay, Ames test, chromosome aberrations test

## Abstract

Hyperforin (HF), a substance that accumulates in the leaves and flowers of *Hypericum perforatum* L. (St. John’s wort), consists of a phloroglucinol skeleton with lipophilic isoprene chains. HF exhibits several medicinal properties and is mainly used as an antidepressant. So far, the antigenotoxicity of HF has not been investigated at the level of primary genetic damage, gene mutations, and chromosome aberrations, simultaneously. The present work is designed to investigate the potential antigenotoxic effects of HF using three different experimental test systems. The antigenotoxic effect of HF leading to the decrease of primary/transient promutagenic genetic changes was detected by the alkaline comet assay on human lymphocytes. The HF antimutagenic effect leading to the reduction of gene mutations was assessed using the Ames test on the standard *Salmonella typhimurium* (TA97, TA98, and TA100) bacterial strains, and the anticlastogenic effect of HF leading to the reduction of chromosome aberrations was evaluated by the in vitro mammalian chromosome aberration test on the human tumor cell line HepG2 and the non-carcinogenic cell line VH10. Our findings provided evidence that HF showed antigenotoxic effects towards oxidative mutagen zeocin in the comet assay and diagnostic mutagen (4-nitroquinoline-1-oxide) in the Ames test. Moreover, HF exhibited an anticlastogenic effect towards benzo(a)pyrene and cisplatin in the chromosome aberration test.

## 1. Introduction

In recent years, the interest in herbal remedies as preventive and therapeutical medicines has been increasing. Some studies have indicated the importance of natural products with potent antigenotoxic activities [1,2,3]. One of the most popular medicinal herbs is the perennial herb *Hypericum perforatum* (*Hypericaceae*), known as St. John’s wort [4]. *H. perforatum* contains numerous biologically active components, including naphtodianthrone derivatives (e.g., hypericin), phloroglucinol derivatives (e.g., hyperforin; HF), flavonoids, procyanidines, tannins, essential oils, phenylpropanes, xanthones, and other hydrosoluble compounds [5]. *H. perforatum* has been used for the treatment of premenstrual syndrome, burns, bruises, eczema, dyspepsia and gastric ulcers, biliary disorders, swellings, inflammations, and anxiety, as well as viral infections [6,7,8]. HF consists of a phloroglucinol skeleton with lipophilic isoprene chains. HF accumulates in the leaves and flowers of St. John’s wort, where it is a major lipophilic constituent. This component has several significant medicinal properties, such as antidepressant, anticarcinogenic, and proapoptotic ones [9]. HF exhibits effective antibacterial activity against multiresistant *Staphylococcus aureus* and other Gram-positive bacteria. However, it has no growth-inhibitory effect on Gram-negative bacteria or *Candida* [9,10]. HF also induces the expression of the cytochrome P-450 isoform CYP3A4 by binding to the pregnane X receptor [11]. On the other hand, it is a potential inhibitor of the major human procarcinogen-activating enzyme, the isoform CYP1A1 [12]. HF induces apoptosis in many tumor cells, by activating caspase-3. In some tumor cells HF also activates either caspase-9 or caspase-8 [13].

So far, the antigenotoxicity of HF has not been investigated at the level of primary genetic damage, gene mutations, and chromosome aberrations, simultaneously. Then, the present work is designed to investigate the potential antigenotoxic effects of non-cytotoxic HF concentrations using different test systems enabling the assessment of primary/transient promutagenic lesions, gene mutations, and chromosome aberrations, using human peripheral lymphocytes, *Salmonella typhimurium* bacterial strains, the human tumor cell line HepG2, and non-cancerous human cells VH10, respectively. To detect the potential antigenotoxic effects, we chose the non-cytotoxic concentrations from the previous experiments in all of three test systems. Owing to the fact that cancer could be linked to or accompanied by depressions, which are often treated with HF, we suggest the results of this research might be useful for therapeutists in their medical practice.

## 2. Results

### 2.1. Cell Viability

**Figure 1:**
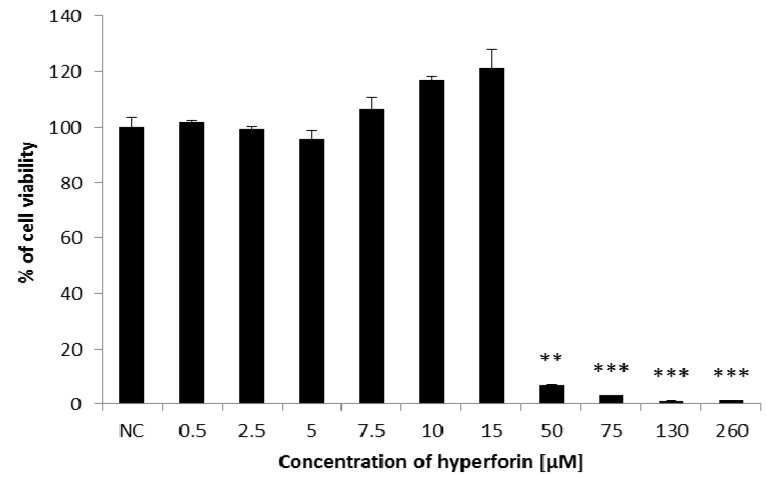
Determination of HF cytotoxic effects towards HepG2 cells using the MTT assay. Data shown are mean ± SD of three repeated experiments. NC = negative control. Comparison with negative control: ** 0.001 < *p* <0.01; *** *p* < 0.001.

The cytotoxicity of HF was detected by the MTT assay, a method for determining the cell viability via the measurement of mitochondrial functionality [14,15]. In 0.5–15 µM concentrations, HF did not reduce the viability of HepG2 cells (Figure 1). However, high concentrations of HF (50–260 µM) significantly reduced the survival of HepG2 cells (Figure 1). The IC50 value was 19.87 µM.

### 2.2. The Alkaline Comet Assay

**Figure 2:**
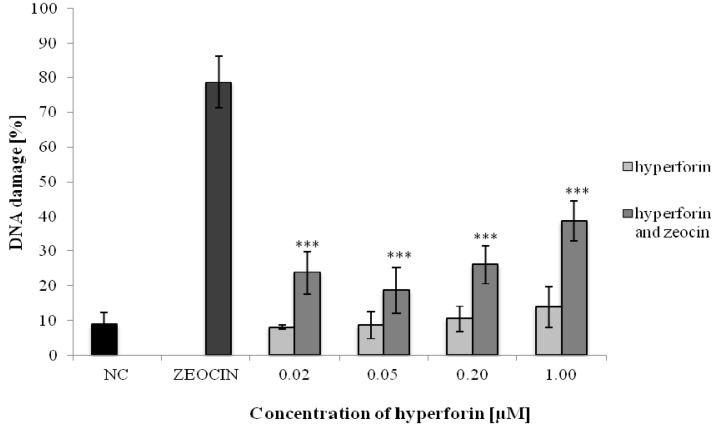
Determination of HF antigenotoxic effects on human lymphocytes using the alkaline comet assay. Data shown are mean ± SD of three repeated experiments. NC = negative control; ZEOCIN = positive control. Comparison with positive control (ZEOCIN): *** *p* < 0.001.

The potential antigenotoxic effects of HF in a concentration range from 0.02 to 1 µM were investigated using the comet assay on human lymphocytes. These concentrations of HF were selected because they did not show any genotoxicity in the preliminary experiments (unpublished data). Pretreatment (24 h) with HF significantly decreased the percentage of damaged DNA, compared to the positive control (Figure 2), due to its antigenotoxic/DNA-protective effects.

### 2.3. The Ames/S. typhimurium Test

**Figure 3:**
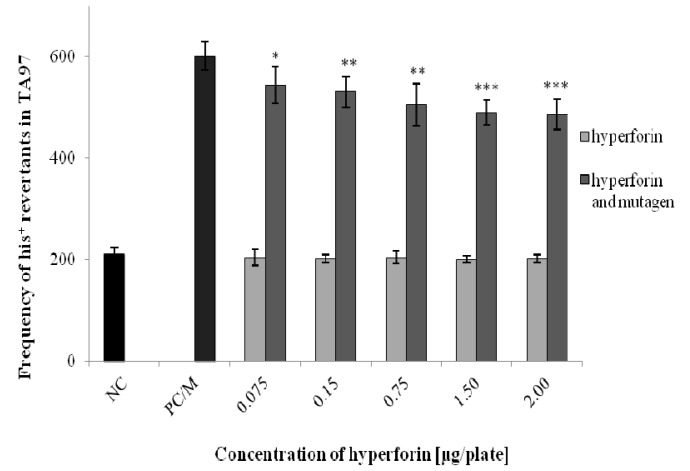
Determination of HF antimutagenic effects using the Ames test—strain *Salmonella typhimurium* TA97. Data shown are mean ±SD of three repeated experiments. NC = negative control; PC/M = positive control = mutagen = (9-aminoacridine). Comparison with positive control (PC/M): * 0.01 < *p* < 0.05; ** 0.001 < *p* < 0.01; *** *p* < 0.001.

**Figure 4:**
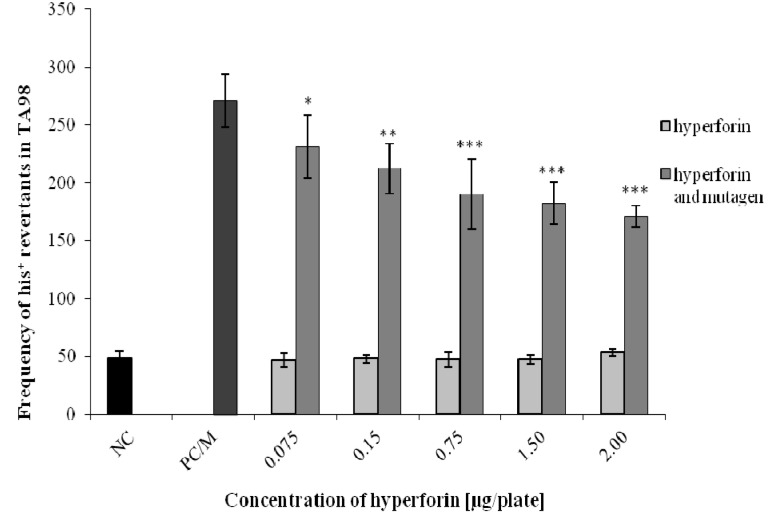
Determination of HF antimutagenic effects using the Ames test—strain *S. typhimurium* TA98. Data shown are mean ±SD of three repeated experiments. NC = negative control; PC/M = positive control = mutagen = (4-nitroquinoline-1-oxide). Comparison with positive control (PC): * 0.01 < *p* < 0.05; ** 0.001 < *p* < 0.01; *** *p* < 0.001.

**Figure 5:**
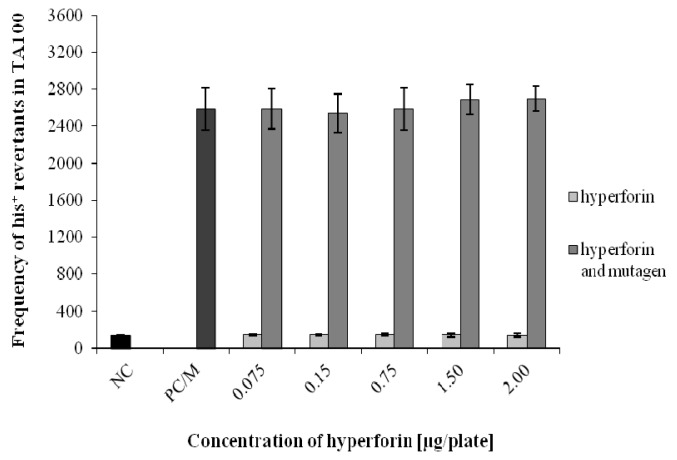
Determination of HF antimutagenic effects using the Ames test—strain *S. typhimurium* TA100. Data shown are mean ±SD of three repeated experiments. NC = negative control; PC/M = positive control = mutagen = (sodium azide).

The Ames test was used to assess the potential antimutagenic effects of HF (at non-mutagenic concentrations 0.075–2 µg/plate) on three bacterial strains of *S. typhimurium* (TA97, TA98, and TA100) (Figure 3, Figure 4 and Figure 5). HF concentrations for detecting the antimutagenic effects were selected based on a range-finding test (unpublished data). The antimutagenic effect of HF was detected using strain TA98 after the combined application of HF with 4-NQO. At all HF concentrations used, we observed a significant decrease in the number of *his^+^* revertants in comparison to the positive control/mutagen (Figure 4). For TA97 we also detected a decrease of *his^+^* revertants (Figure 3). Moreover, the antimutagenic effect of HF was not observed in the strain TA100 (Figure 5). The same results were obtained when an S9 mix, together with a diagnostic mutagen 2-aminoflourene (50 µg/plate), was used for all three bacterial strains (unpublished data).

### 2.4. In Vitro Mammalian Chromosome Aberration Test 

For determination of the HF anticlastogenic effect against B(a)P, HepG2 cells were treated with B(a)P (1.7 µM) after being pretreated with two non-clastogenic concentrations of HF (0.75 and 1.5 µM), selected in preliminary experiments (unpublished data). For both concentrations of HF a significant difference in the amount of aberrant metaphases and chromosome aberrations, in comparison to the positive control (HepG2 cells treated only with benzo(a)pyrene), was observed (Table 1). We have revealed a significant decrease in the main cytogenetic parameters as well.

In HepG2 cells treated with 0.5 µM cisPt, after being pretreated (24 h) with two concentrations of HF (0.75 and 1.5 µM), a significant decrease in the amount of aberrant metaphases and chromosome aberrations was observed (Table 2). The pretreatment (24 h) of VH10 cells with different concentrations of HF and subsequent treatment with cisPt (0.75 µM) showed a significant decrease in the amount of aberrant metaphases and chromosome aberrations, similar to the HepG2 cells (Table 2).

In our experiments evaluating the potential anticlastogenic activity of HF using the cytogenetic method, we observed that HF exhibited anticlastogenic effects against both the indirect mutagen B(a)P and direct mutagen cisPt.

## 3. Discussion

HF is one of the major components of *H. perforatum* (St. John’s wort), responsible for many health-promoting activities [16]. Since it has been demonstrated that HF exhibits antioxidant properties [17], it was a high probability that it could exhibit also antigenotoxic activities. To the best of our knowledge, this was the first time the potential antigenotoxicity of HF was examined at three different levels simultaneously; primary/promutagenic DNA lesions, gene mutations, and chromosome aberrations.

In the assessment of HF cytotoxic activity towards the HepG2 cell line, we found out that lower concentrations of HF were not cytotoxic. However, high concentrations of HF significantly reduced the cell viability. Komoroski et al. [18] observed that even lower concentrations of HF (2.5 and 5 µM) caused a decrease in mitochondrial activity in primary hepatocytes, which led to the reduction of cell vitality. The discrepancy in our results could be explained by the fact that different cell lines were used; for example, the HepG2 cell line in our experiments versus the primary hepatocytes used by Komoroski et al. [18].

Consequently, we have focused on detecting the potential antigenotoxic effects of HF towards the oxidative mutagen zeocin at the level of transient/primary DNA lesions (e.g., single-strand breaks—SSBs), applying the comet assay. HF showed high antigenotoxic activity against zeocin.

Zeocin, as a member of the bleomycin/phleomycin family, isolated from *Streptomyces verticullus,* is used clinically as an antitumor drug [19]. Radiomimetic zeocin induces an oxidative damage to DNA [20] that can lead to the formation of SSBs and double-strand breaks (DSBs), as results of the oxidative stress [21,22]. HF may protect DNA against DNA break formation by acting as an antioxidant. Antioxidant properties, based mainly on the free radical scavenging activity of HF, were pointed out in our previous study [17].

Two major genetic changes, gene mutations and chromosome aberrations are often implicated in the activation of oncogenes or inactivation of tumor suppresor genes in the process of malignant transformation [23]. The detection of the DNA-protective effect of HF against the induction of DNA SSBs and DSBs by zeocin was followed by establishing its potential antimutagenic and anticlastogenic effects at the level of gene mutations and chromosome aberrations, using another experimental model.

In the Ames test, we investigated the potential antimutagenic effects of HF on three bacterial strains of *S. typhimurium* (TA97, TA98, and TA100). The strain TA97 enables the detection of frameshift mutagens [24]. 9-aminoacridine, which was used as diagnostic mutagen for this strain, interacts with GC base-pairs and induces frameshift mutations due to DNA intercalation [25]. A diagnostic mutagen for the strain TA98, 4-nitroquinoline-1-oxide, is metabolized into 4-acetoxy amino quinoline-1-oxide. It forms covalent adducts to C8 or N2 of deoxyguanine and N6 of deoxyadenine in DNA. It also produces oxidative damages and DNA SSBs, which may lead to mutations [26]. For the strain TA100, we used sodium azide as a diagnostic mutagen that can be metabolized to azidoalanine and causes base-pair substitutions [27]. Using the strain TA98, the antimutagenic activity of HF was revealed. The strain TA97 also exhibited a decrease in the number of *his^+^* revertants. This decrease was statistically significant, but such a reduction of revertants is rather small to be biologically significant. It is known that strains TA97 and TA98 may respond to the same (frameshift) mutagens [28]. This could be also the reason why these strains respond similarly to some bioactive compounds (e.g., HF), exerting DNA-protectivity [17].

Subsequently, the anticlastogenic ability of HF was studied using two differently acting mutagens; benzo(a)pyrene (B(a)P) and cisplatin (cisPt). B(a)P, as a polycyclic aromatic hydrocarbon, possesses the carcinogenic, mutagenic, and teratogenic activities observed in various species and tissues [29,30]. B(a)P is one of the compounds responsible for the smoking-related human lung cancer, as B(a)P-induced DNA adducts are formed preferentially in the mutational hot spots of the *TP53* gene [31]. B(a)P toxicity and mutagenicity is mediated by the reactive intermediate compounds. These intermediates are formed during B(a)P metabolism, by the microsomal cytochrome P-450 enzymes, such as CYP1A and CYP1B. The reactive intermediates can bind to the DNA and form covalent adducts, which cause carcinogenesis [32]. It was observed that the human enzymes CYP1, mainly CYP1A1, CYP1A2, and CYP1B1, play the key role in the activation of B(a)P to B(a)P 7,8-diol-9,10-epoxide (ultimate mutagen/carcinogen) [33]. Other human cytP-450 enzymes, such as CYP2C9 and CYP3A4, are also important for the metabolic activation of B(a)P [34]. Changes in cytP-450 activity may affect the metabolism and clearance of various drugs [35]. Paine et al. [36] observed that among all cytochromes P-450 present in the liver, CYP3A, CYP2C, and CYP1A1 occurred at highest concentrations. The cell line HepG2, which is derived from human liver tumors, is characterized by many xenobiotic metabolizing activities because the stable expression of the human CYP subtypes was detected in the HepG2 cells [37]. This cell line is useful for employment in experiments aimed at the prediction of the cytotoxicity and metabolism of different chemicals occurring in the human liver [38]. In the HepG2 cell line pretreated with HF, we have revealed a significant decrease of structural aberrations, when applying B(a)P. Therefore, we can conclude that HF exhibited anticlastogenic activity against B(a)P, probably due to inactivation of HepG2 microsomal cytochrome P-450 enzymes. Schwarz et al. [12] also examined the effects of various components of *H. perforatum*, including hyperforin. They found that *H. perforatum* extract inhibited CYP1A1-catalyzed 7,8-diol benzo(a)pyrene epoxidation, a terminal reaction leading to the ultimate carcinogenic product (+) benzo(a)pyrene-7,8-dihydrodiol-9,10-epoxide. HF served as a competitive inhibitor of CYP1A1 [12]. Obach [39] found that HF was also an inhibitor of other enzymes, mainly being a competitive inhibitor of CYP2C9 and a non-competitive inhibitor of CYP2D6. Based on the above mentioned observations we suggest that the inhibition of these enzymes by HF might be the reason for its anticlastogenic activity towards B(a)P, observed in the HepG2 cell line.

In experiments aimed at the detection of the HF anticlastogenetic effect, both cell lines (HepG2 and VH10) were treated with a direct mutagen, cisPt, which belongs to the first-line chemotherapeutic agents for the treatment of cancer [40,41,42]. The cell resistance against cisPt could be caused by the increased drug detoxification, based on P-glycoprotein, or by the excessive DNA repair. HF regulates the expression of P-glycoproteins and induces their activities [43,44]. P-glycoprotein is a membrane protein, which is responsible for the mechanism of resistance of cancer cells during anti-tumor treatments [45]. Its physiological role is to protect cells against exogenous and endogenous cellular toxins. P-glycoprotein expression is high in the colon, intestine, kidney, pancreas, and liver [46,47]. These tumors are primarily resistant to chemotherapy. P-glycoprotein induces changes in cell regulatory pathways that lead to the partial loss of sensitivity to cisPt [47].

The primary reason for developing structural aberrations is always caused by the occurrence of chromatid or chromosomal breaks. In our study, we observed that cisPt caused chromosome aberrations, but when both cell lines were treated with cisPt after pretreatment with HF, anticlastogenic activity was revealed. Chromosome aberrations are caused by the incorrect repair of DSBs, which can be repaired by two mechanisms; non-homologous end joining (NHEJ) and homologous recombination [48]. CisPt induces DNA interstrand crosslinks, which represent blocks in the DNA metabolic processes, mainly in the DNA replication. These DNA damages must be repaired for cell survival. CisPt-DNA adducts are repaired especially by nucleotide excision repair (NER) [49]. However, Wu et al. [50] found that mismatch repair might also participate in the error-free processing of DNA interstrand crosslinks in human cells.

The anticlastogenic effect of HF towards cisPt could be explained by its increased detoxification, based on P-glycoprotein, or by excessive DNA repair stimulation. However, it may be possible that the anticlastogenic effect of HF towards cisPt is also caused by the fact that HF is an antioxidant [17].

HF is a naturally occurring substance with significant healing effects that has a great potential to become an important part of various medicinal preparations. Based on its antigenotoxic activities, HF should contribute to the decrease of a human environmental health risk. However, according to our results we do not recommend treatment of depression with HP in oncological patients treated with cisPt as a therapeutic agent. The effectiveness and overall potency of such therapy could be substantially reduced.

## 4. Materials and Methods

### 4.1. Materials

PBS (Phosphate buffered saline, Oxoid Limited, Hampshire, UK), DMSO (dimethyl sulfoxide, PAN-Biotech GmbH, Aidenbach, Germany, CAS No. 67-68-5), Histopaque 1077 (Sigma-Aldrich, Bratislava, Slovakia, CAS No. 97639-11-7), Wiliams medium (PAN-Biotech) with 10% fetal bovine serum (PAN-Biotech GmbH, Aidenbach, Germany, CAS No. 9048-46-8), Gentamycin (PAN-Biotech GmbH, Aidenbach, Germany, CAS No. 1405-41-0), Eagle medium (PAN-Biotech GmbH, Aidenbach, Germany) with 10% fetal bovine serum (PAN-Biotech GmbH, Aidenbach, Germany, CAS No. 9048-46-8), Hyperforin (AppliChem GmbH, CAS No. 238074-03-8), MTT (3-(4,5-Dimethyl-2-thiazolyl)-2,5-diphenyl-2H-tetrazolium bromide, Sigma-Aldrich, Bratislava, Slovakia, CAS No. 298-93-1), Zeocin (Invivogen, San Diego, CA, USA, CAS No. 11006-33-0), Low melting point agarose (LMP, Sigma-Aldrich, Bratislava, Slovakia, CAS No. 39346-81-1), Normal melting point agarose (NMP, Sigma-Aldrich, Bratislava, Slovakia, CAS No. 9012-36-6), NaCl (Slavus, Bratislava, Slovakia, CAS No. 7647-14-5), Na_2_EDTA (Sigma-Aldrich, Bratislava, Slovakia, CAS No. 139-33-3), Tris–HCl (Sigma-Aldrich, Bratislava, Slovakia, CAS No. 77-86-1), Triton X-100 (Sigma-Aldrich, Bratislava, Slovakia, CAS No. 9002-93-1), Ethidium bromide (Sigma-Aldrich, Bratislava, Slovakia, CAS No. 1239-45-8), 9-aminoacridine (Serva, Heidelberg, Germany, CAS No. 52417-22-8), 4-nitrocquinoline 1-oxide (Serva, Heidelberg, Germany, CAS No. 56-57-5), Sodium azide (Serva, Heidelberg, Germany, CAS No. 26628-22-8), 2-aminofluorene (Serva, Heidelberg, Germany, CAS No. 153-78-6), Benzo(a)pyrene (Sigma-Aldrich, Bratislava, Slovakia, CAS No. 50-32-8), Cisplatin (cisplatinum(II)diamine dichloride, ICN Biomedicals Inc., Aurora, OH, USA, CAS No. 15663-27-1), and H_2_O_2_ (Hydrogen peroxide, Sigma-Aldrich, CAS No. 7722-84-1). All commercial and synthesized chemicals had a purity of at least 95%.

### 4.2. Experimental Cells

#### 4.2.1. Human Lymphocytes

In the alkaline comet assay human lymphocytes were used. They were isolated from peripheral blood obtained by the finger prick method. To 1 mL of ice cold 1× PBS, 40 μL of peripheral blood was added and allowed to stand on ice for 30 min. Lymphocytes were separated from whole blood samples by standard centrifugation with 100 μL of the Histopaque medium (Sigma-Aldrich). After the centrifugation, 200 μL of isolated cells were resuspended in 1 mL of 1× PBS buffer and re-centrifuged.

#### 4.2.2. Bacterial Strains of *S. typhimurium*

Three bacterial strains of *S. typhimurium—*TA97, TA98, and TA100 were used in the Ames test. They were obtained from the Czech Collection of Microorganisms (Brno, Czech Republic).

#### 4.2.3. Human Cell Line HepG2

The cell line HepG2 was used in both in the MTT test and the in vitro mammalian chromosome aberration test. Cells were derived from human liver tumors, originally established by Dr. B.B. Knowles (Wistar Institute of Anatomy and Biology, Philadelphia, PA, USA) and kindly provided by A. Collins (Department of Nutrition, University of Oslo, Oslo, Norway). The cell line HepG2 is capable of activating many indirect mutagens by its expression of the cytochrome P-450 superfamily members (CYP1A1, CYP1A2, CYP2C9, CYP3A4, CYP2C19, CYP2A6, CYP2B6, CYP2C8, CYP2D6, and CYP2E1) [37]. This cell line is characterized by an aneuploid number of chromosomes. The HepG2 cell line was used at passage number 21. Cells were cultured in Wiliams medium (PAN-Biotech GmbH) with 10% fetal bovine serum (PAN-Biotech GmbH). The Williams medium was supplemented with antibiotic gentamycin (50 μg/mL) (PAN-Biotech GmbH). Cells were cultured in plastic Petri dishes (Ø = 60 mm) under CO2/air (5%:95%) at 37 °C, as described by Miadokova et al. [51].

#### 4.2.4. Human Cell Line VH10

The VH10 cell line used in the in vitro mammalian chromosome aberration test was derived from human non-malignant diploid fibroblasts, isolated from the foreskin of a healthy boy [52]. The cell line was obtained from D. Slameňová (Cancer Research Institute, Bratislava, Slovakia). The VH10 cell line was used at passage number 12. The VH10 cells were cultured in MEM Eagle medium (PAN-Biotech GmbH, Aidenbach, Germany) with 10% fetal bovine serum and supplemented with antibiotic gentamycin (50 μg/mL). Cells were cultured in plastic Petri dishes (Ø = 60 mm) under CO_2_/air (5%:95%) at 37 °C.

### 4.3. Cell Viability (MTT Test)

The cytotoxic effect of HF was determined by the MTT assay [14,15]. After 24 h of cultivation, the HepG2 cells, placed in 96-well culture plates, were treated with HF (0.5; 2.5; 5; 7.5; 10; 15; 50; 75; 130; 260 µM). 42 h after treatment with HF, the cells were incubated with 50 µL of MTT (1 mg/mL) and left in the dark at 37 °C for an additional 3 h. Thereafter, the medium was removed, and replaced by 200 µL of DMSO. The absorbance was measured at 540 and 690 nm using an xMarkTM Microplate Spectrophotometer (Bio-Rad Laboratories, Inc., Berkeley, CA, USA). The concentration of HF that inhibited cell survival to 50% (IC50) was calculated by Calcusyn software, version 1.1, Biosoft.

### 4.4. Comet Assay

This assay was selected for detecting the potential antigenotoxic effect of HF towards zeocin on the human lymphocytes. The assay was performed according to Lorenzo et al. [53] and Horvathova et al. [54]. Briefly, human lymphocytes were suspended in 1% low melting point agarose (LMP) in PBS and placed on slides pre-coated with 1% normal melting point agarose (NMP). Slides with embedded lymphocytes were incubated for 1 h in HF solution (0.02; 0.05; 0.2; 1 µM). Afterwards, the solution of HF was rinsed off with PBS and treated with zeocin (4.5 μM) for 2 min. Afterwards, the zeocin solution was rinsed off with PBS. Positive controls were treated only with zeocin (4.5 µM) for 2 min. For the negative control, cells were left untreated in PBS for 1 h. Two slides from each sample were lysed in a lysis solution: 2.5 M NaCl (Centralchem, Bratislava, Slovakia), 100 mM Na_2_EDTA (Sigma-Aldrich, Bratislava, Slovakia), 10 mM Tris–HCl, pH 10 (Sigma-Aldrich, Bratislava, Slovakia), and 1% Triton X-100 (Sigma-Aldrich, Bratislava, Slovakia) for 1 h at 4 °C. DNA from the lymphocytes embedded on the microscope slides was unwound in electrophoresis solution for 20 min. Electrophoresis was performed at 4 °C, 25 V, and 270—300 mA for 30 min. After electrophoresis, the slides were neutralized in PBS (5 min) and ddH2O (5 min) at 4 °C. Then the slides were left to dry for 12 h. Finally, the slides were stained with 20 µg/mL of ethidium bromide (Sigma-Aldrich, Bratislava, Slovakia), and, in each sample, 100 random nucleoids were scored at magnification 400× using a fluorescence microscope Olympus BX 51. The DNA damage was evaluated using the Comet visual computer software. DNA damage was expressed as the percentage of DNA in the tail (% tail DNA) [55]. The experiments were repeated at least 3 times and statistical analysis was performed using Student’s *t*-test.

### 4.5. Ames/S. typhimurium Test

The assay was selected for the detection of the potential antimutagenic effect of HF. The Ames assay was conducted according to the method modified by Maron and Ames [28]. In the Ames test, three bacterial strains of *S. typhimurium* (TA97, TA98, and TA100) were used. These strains are deficient in histidine and biotin, and, at the same time, they are deficient in excision repair. Prior to each experiment, the quality of each bacterial strain was checked similarly, as described by Berg et al. [56]. The assay was carried out in sterile test tubes containing 100 µL (approximately 1.10^8^ cells/mL) of overnight bacterial culture (overnight cultivated in 50 mL of LB medium) and 2.5 mL of the top-agar (with traces of histidine and biotin). In the case of metabolic activation, S9 mix was used to activate a promutagen 2-aminofluorene (50 µg/plate, Serva, Heidelberg, Germany). Then distilled water (as a negative control; NC), selected non-mutagenic concentrations of HF (0.075; 0.15; 0.75; 1.5; 2.0 µg/plate), and HF combined with positive mutagens were added. As positive controls (PC), the following diagnostic/direct mutagens were used: 9-aminoacridine (50 µg/plate, Serva, Heidelberg, Germany) for TA97; 4-nitroquinoline-1-oxide (20 µg/plate, Serva, Heidelberg, Germany) for TA98; and sodium azide (50 µg/plate, Serva, Heidelberg, Germany) for TA100. The contents of each test tube were mixed well and then poured into a minimal glucose agar plate (three Petri dishes per each HF concentration/control). After 48 h of cultivation at 37 °C, *his^+^* revertants were counted. All experiments were conducted in triplicate. The data obtained were then analyzed using Student’s *t*-test.

### 4.6. In Vitro Mammalian Chromosome Aberration Test

The assay was selected for the detection of the potential anticlastogenic effect of HF. HepG2 and VH10 cells were seeded into Petri dishes (Ø = 60 mm; HepG2 inoculum of 1 × 10^6^ cells/dish and VH10 inoculum of 3.8 × 10^5^ cells/dish) for 2 h. Then, HF non-clastogenic concentrations (0.75 or 1.5 µM) were added and the cultures were incubated for 24 h. These concentrations of HF were chosen based on preliminary experiments (range-finding tests). Afterwards the medium was replaced with fresh medium alone or with fresh medium containing mutagens B(a)P (1.7 μM for HepG2 cells) or cisPt (0.5 μM for HepG2 cells and 0.75 μM for VH10 cells), and the HepG2 cells were cultivated further for 42 h and VH10 cells for 36 h, which approximately corresponds to 1.3 cell cycles of HepG2 and VH10 cells. Three hours before the end of the cultivation, colchicine (0.75 µg/mL) was added. Before karyological processing, the cells were counted. Slides were prepared by the standard air-drying method and were stained with 2% aqueos Giemsa-Romanowski solution for 10 min. The indirect mutagen benzo(a)pyrene (B(a)P; Sigma-Aldrich, Bratislava, Slovakia) (1.7 and 10 μM) and the direct mutagen cisplatin (cisPt; cisplatinum(II)diamine dichloride, ICN Biomedicals Inc.) (0.5 and 0.75 μM) were used as a positive control (PC).

Chromosome aberrations were evaluated by a microscopic examination. For each sample, 100 metaphases were analyzed. We focused on the following structural aberrations; breaks, chromatid, and iso-chromatid, and exchanges, including dicentrics, rings, tri-radials and quadri-radials. The results were evaluated statistically on the basis of the difference between two relative values. The percentage of aberrant metaphases and the total number of chromosome aberrations was compared to solvent control (SC), or clastogenic effect, and positive control (PC), or anticlastogenic effect. Since the genetic significance of gaps is not clearly understood, they were not included in the assessment of chromosomal damage and thus were not evaluated statistically [51].

## Figures and Tables

**Table 1:** Determination of HF anticlastogenic effect towards benzo(a)pyrene using the chromosome aberration test on HepG2 cells.

HF/B(a)P Concentration (µM)	Number of Aberrant Metaphases	Number of Chromosome Aberrations									Total Number of CA
		Chromatid		Isochromatid		Exchange					
		g	b/f	g	b/f	ring	dic	qr	tr	dmin	
HepG2											
IC	4	1	3	-	-	-	-	-	-	1	4
SC	3	1	3	-	-	-	-	-	-	-	3
HF 0.75	2	1	2	-	-	-	-	-	-	-	2
HF 1.5	2	-	2	-	-	-	-	-	-	-	2
PC B(a)P 1.7	35 ***	-	33	1	5	-	1	-	-	-	39 ***
HF 0.75 + B(a)P 1.7	8 ^###^	1	7	-	1	-	-	-	-	-	8 ^###^
HF 1.5 + B(a)P 1.7	8 ^###^	1	8	-	-	-	1	-	-	-	9 ^###^

IC—intact control; SC—solvent control (DMSO); HF—hyperforin; PC B(a)P—positive control (benzo(a)pyrene); CA—chromosome aberrations; g—gap; b/f—break and/or fragment; dic—dicentric; qr—quadriradial; tr—triradial. Comparison with solvent control (SC): *** *p* < 0.001. Comparison with positive control (PC): ^###^*p* < 0.001.

**Table 2:** Determination of HF anticlastogenic effect towards cisplatin using the chromosome aberration test on HepG2 and VH10 cells.

HF/cisPt Concentration (µM)	Number of Aberrant Metaphases	Number of Chromosome Aberrations									Total Number of CA
		Chromatid		Isochromatid		Exchange					
		g	b/f	g	b/f	ring	dic	qr	tr	dmin	
**HepG2**											
IC	2	1	2	1	-	-	-	-	-	-	2
SC	3	1	2	-	-	-	1	-	-	-	3
HF 0.75	1	1	1	-	-	-	-	-	-	-	1
HF 1.5	3	-	2	-	1	-	-	-	-	-	3
PC cisPt 0.5	38 ***	2	36	-	8	-	2	1	-	-	47 ***
HF 0.75+ cisPt 0.5	10 *^,###^	2	5	-	4	-	2	-	-	-	11 *^,###^
HF 1.5 + cisPt 0.5	14 **^,###^	2	10	-	3	-	1	-	-	-	14 **^,###^
**VH10**											
IC	2	-	2	-	-	-	-	-	-	-	2
SC	2	1	1	1	-	-	1	-	-	-	2
HF 0.75	1	-	1	-	-	-	-	-	-	-	1
HF 1.5	1	-	-	-	-	-	1	-	-	-	1
PC cisPt 0.75	33 ***	2	26	-	9	-	-	4	5	-	44 ***
HF 0.75 + cisPt 0.75	12 **^,###^	1	8	-	3	-	1	1	1	-	14 ***^,###^
HF 1.5 + cisPt 0.75	11 **^,###^	3	8	-	-	-	1	-	2	-	11 **^,###^

IC—intact control; SC—solvent control (PBS); HF—hyperforin; PC cisPt—positive control (cisplatin); CA—chromosome aberrations; g—gap; b/f—break and/or fragment; dic—dicentric; qr—quadriradial; tr—triradial. Comparison with solvent control (SC): * 0.01 < *p* < 0.05; ** 0.001 < *p* < 0.01; *** *p* < 0.001. Comparison with positive control (PC): ^###^*p* < 0.001.
